# Gender Differences in Depression and Quality of Life in Current and Abstinent Ketamine Users

**DOI:** 10.3390/ijerph18189567

**Published:** 2021-09-10

**Authors:** Peng-Wei Wang, Cheng-Fang Yen, Hung-Chi Wu, Chih-Yao Hsu, Yu-Yi Yang

**Affiliations:** 1 Department of Psychiatry, School of Medicine, College of Medicine, Kaohsiung Medical University, Kaohsiung 80708, Taiwan; chfaye@kmu.edu.tw (C.-F.Y.); iugyyy@gmail.com (Y.-Y.Y.); 2 Department of Psychiatry, Kaohsiung Medical University Hospital, Kaohsiung 80708, Taiwan; 3 Departments of Addiction Science, Kai-Suan Psychiatric Hospital, Kaohsiung 80708, Taiwan; wuhcmail@gmail.com (H.-C.W.); oldtype@gmail.com (C.-Y.H.)

**Keywords:** ketamine use disorder, quality of life, depression and gender

## Abstract

Ketamine use has become of increasing concern because it has spread in many parts of the world during the past few years. Substance users usually have depression and a lower quality of life (QoL). The aim of this study was to explore depression and QoL in ketamine users, and to further examine the role of gender in relation to differences in depression and QoL in ketamine users. This study recruited 204 current ketamine users, 102 abstinent ketamine users and 102 healthy controls. The demographic data, severity of depression and QoL were recorded. Analysis of Variance (ANOVA) was employed to compare the associations of ketamine use status with depression and QoL. Gender differences were examined by moderator analysis. The current ketamine users with and without ketamine use disorder, in addition to the abstinent ketamine users with ketamine use disorder, have more severe depression and a lower QoL than healthy controls. There were significant gender differences in depression and QoL in abstinent ketamine users with ketamine use disorder. Ketamine users have more severe depression and a lower QoL. In particular, depression and a lower QoL are still prominent in abstinent ketamine users. The gender differences in depression and QoL are significant in abstinent ketamine users.

## 1. Introduction

Ketamine is an important medicine used in anesthesia; however, non-medical use of ketamine has spread in many parts of the world during the past few years [1]. Rajesh et al. showed that people with chronic non-dependent ketamine use, which means the non-medical use behavior does not meet criteria of ketamine use disorder by DSM 5 [2], have impaired prefrontal dopaminergic transmission, a circuit critically involved in working memory and executive function [3]. Furthermore, non-dependent ketamine use may result in neurobehavioral abnormality and physical illnesses [4,5]. In addition, ketamine dependence, which means individuals with ketamine use disorder, is exhibited as brain structure impairment that develops as significant cortical atrophy in the frontal, parietal, or occipital cortices [6]. The non-dependent ketamine use may be associated with the development of dependent ketamine use [7]. These results indicated that both non-dependent and dependent ketamine use are important health issues.

The results of a study that included 6355 drug-dependent patients from 41 sites showed that 44% of these drug-dependent individuals also had a lifetime history of major depression [8]. Another analysis of more than 4000 drug-dependent patients revealed strong associations of both alcohol and drug use disorders with depression [9]. In addition, substance use may induce depression. A study focused on alcohol use disorder revealed that the rate of alcohol-induced major depressive disorder ranged from 8% to 53%, and the rate of alcohol-induced depressive symptomatology ranged from 16% to 59% [10]. Mohamed et al. showed that drug users with greater substance use are more likely to experience more severe depressive symptoms [11]. Therefore, depression is a significant mental health problem in people with substance use disorders.

Drug use can damage individuals’ physical and mental health, job performance, and social adaptation [12,13]. Studies on alcohol and illicit substance use showed that life quality is poor among substance users [14,15,16]. In addition, greater improvement in the quality of life (QoL) is associated with less drug use in people receiving drug treatment [17]. Given the chronic and relapsing nature of substance use disorder and the various life domains it can affect, there is growing evidence to show that QoL can be a useful assessment tool and outcome measure in patients treated for substance use disorders [18,19].

Patients at all stages of substance use may experience concerns about multiple areas of function that can impair QoL and cause depressive symptoms [20,21]. Few studies have explored the associations of status of ketamine use with the level of QoL and severity of depression. Studies have shown that female subjects are much more likely to be diagnosed with depression and have more severe depressive symptoms than males [22,23,24]. Coelho et al. also showed that the severity of depression is higher in female substance users [25]. In addition, previous studies have indicated that women have a lower QoL than men [26,27]. A study of cannabis users revealed that the female gender and severity of cannabis use are associated with a reduced QoL [28]. However, research into gender differences in ketamine users of varying status is scarce. The present study aimed to explore (1) the severity of depression and QoL, and (2) gender differences in the severity of depression and QoL in ketamine users of differing status. Therefore, we drew up the following hypotheses: (1) ketamine users have a lower QoL and more severe depressive symptoms than healthy people; (2) dependent ketamine users may be associated with more severe depressive symptoms and a lower QoL than non-dependent ketamine users; and (3) the severity of depression and QoL differ in ketamine users of different genders.

## 2. Materials and Methods

### 2.1. Participants

All participants, except the abstinent ketamine users, were recruited from the community. The abstinent ketamine users were recruited from four control environments where access to the drug is limited and which do not provide any treatment for drug use. The community ketamine users met the following criteria: (1) current ketamine use with and without ketamine use disorder which is diagnosed by DSM 5 [2]; (2) no comorbid other substance use except tobacco use; (3) no mental illness, including schizophrenia, major depressive disorder or bipolar disorder; and (4) no physical illnesses. The abstinent ketamine users with ketamine use disorder met the following criteria: (1) ketamine use disorder; (2) no comorbid other substance use except tobacco use; (3) currently had stayed in control environments for more than 3 months; (4) no comorbid mental illness, including schizophrenia, major depressive disorder or bipolar disorder; and (5) no physical illnesses. The inclusion criteria for the healthy controls were: (1) no substance use disorder or mental illness; (2) no physical illnesses; and (3) age-, gender- and education-matched to the ketamine users. At first, all eligible participants were interviewed by psychiatrists to evaluate whether or not participants had ketamine use disorder by DSM 5 [2] and fulfilled the other inclusion criteria. The dependent ketamine users were participants with ketamine use disorder. Participants with ketamine use, but who did not meet the criteria of ketamine use disorder by DSM 5, were classified into non-dependent ketamine users. Then, participants who met the inclusion criteria underwent interviews to collect demographic data, data on depression and QoL. The study protocol was approved by the institutional review board of Kaohsiung Medical University. All participants gave written informed consent to participate in this research.

### 2.2. Assessments

#### 2.2.1. Chinese Version of the Center for Epidemiological Studies-Depression Scale (CES-D)

The Chinese version of the CES-D has been used to study depression in Taiwan for many years [29]. Subjects were asked how often they had experienced each symptom during the past week. Response categories included: (0) rarely or none of the time (less than 1 day); (1) some or a little of the time (1–2 days); (2) occasionally or a moderate amount of the time (3–4 days); or (3) most or all of the time (5–7 days). The values of these response categories were reversed for the four positive-affect items. The total score ranged from 0 to 60, and a higher CES-D total score indicated more severe depression.

#### 2.2.2. Taiwan Version of the Brief Version of the World Health Organization Quality of Life Instrument (WHOQOL-BREF)

The Taiwan version of the WHOQOL-BREF consisted of 28 items, including 26 standard items from the original WHOQOL-BREF and two Taiwanese national items. The values for Cronbach’s alpha of the original version were acceptable, i.e., physical domain 0.82, psychological domain 0.81, and environmental domain 0.80, although marginally for the social relationships domain at 0.68 [30]. The internal consistency (Cronbach’s alpha) coefficients of the Taiwan version ranged from 0.70 to 0.77 for the four domains. The content validity coefficients were in the range of 0.53 to 0.78 for item–domain correlations and 0.51 to 0.64 for inter-domain correlations (all *p* < 0.01) [31].

### 2.3. Data Analysis

The chi-squared (χ^2^) test was used to analyze discrepancies in categorical variables among groups. The gender differences in depression and QoL in all groups were analyzed using the t test. Differences in age, education level, severity of depression and QoL were examined using analysis of variance (ANOVA). If there was a significant group difference according to ANOVA, group comparisons were performed using post hoc analysis. We used a regression model to explore the associations of severity of depression and QoL with different types of ketamine use behavior, including healthy controls, abstinent dependent users, current non-dependent users and current dependent users, after controlling gender, age and education. To explore whether the gender effects on the severity of depression and QoL differed from the healthy controls, we added an interaction between gender and type of ketamine use behavior into the regression model [32]. The sequential Bonferroni procedure was used to adjust for multiple comparisons [33].

## 3. Results

### 3.1. Participant Variables and Level of QoL and Depression

Four-hundred and eight people were enrolled. Age, education and gender did not differ among the groups (Table 1). There were significant group differences in the severity of depression and each domain of QoL. The current ketamine users with ketamine disorder had the most severe depression and the lowest level for each domain of QoL of the three groups. Both the current ketamine users without ketamine use disorder and the abstinent ketamine users with ketamine use disorder had significantly more severe depressive symptoms and lower levels in each domain of QoL than the healthy controls. The abstinent ketamine users with ketamine use disorder did not significantly differ in terms of the severity of depression, social domain of QoL and environmental domain of QoL from the current ketamine users without ketamine use disorder. However, the abstinent ketamine users with ketamine use disorder had better physical and psychological QoL scores than the current ketamine users without ketamine use disorder.

### 3.2. Gender Difference in the Level of QOL and Depression

The healthy male subjects had fewer depressive symptoms and a better QoL in each domain than the healthy female controls (Table 2). Meanwhile, the abstinent male ketamine users with ketamine use disorder also had less severe depressive symptoms and a better QoL in each domain than the abstinent female ketamine users with ketamine use disorder. In the current ketamine users with ketamine use disorder, there were no significant gender differences in terms of depressive symptoms or QoL. The male current ketamine users without ketamine use disorder had a significantly higher level of QoL in the environmental domain. However, there were no gender differences in terms of depressive symptoms or the three other domains of QoL in the current ketamine users without ketamine use disorder.

Both gender and status of ketamine use were significantly associated with the severity of depression and QoL in the physical and psychological domains after controlling for the effects of age and education level (Table 3). Compared with the healthy controls, the current and abstinent ketamine users with ketamine use disorder had low social and environmental domain QoL scores after controlling for the effects of age and education. However, the associations between gender and QoL in the social and environmental domains were not significant after controlling for the effects of age and education.

The interactions between gender and the status of ketamine use with depressive symptoms were significant in the abstinent ketamine users and current ketamine users without ketamine use disorder (Table 4). Regarding QoL in the physical, psychological and environmental domains, significant interactions were observed in the current ketamine users both with and without ketamine use disorder. In addition, the interaction between gender and the status of ketamine use on the social domain of QoL was only significant in the current ketamine users with ketamine use disorder.

## 4. Discussion

There were several important findings of the present study. First, the abstinent and non-abstinent ketamine users had more severe depressive symptoms and a poorer QoL than the healthy controls. Second, the severity of depressive symptoms and QoL differed by gender in the abstinent ketamine users with ketamine use disorders. In the current ketamine users, the gender difference was only significant in the environmental domain of QoL in those without ketamine use disorder. Third, the interaction between gender and ketamine use on the severity of depression and QoL varied by the status of ketamine use.

Our results showed that the subjects who used ketamine currently with and without ketamine use disorder suffered from more severe depressive symptoms than the healthy controls. In addition, the current ketamine users with ketamine use disorder were more severely depressed than the current ketamine users without ketamine use disorder. This supported the idea that currently dependent individuals are associated with more severe depressive symptoms than currently non-dependent individuals. The relationship between depression and ketamine use may be bidirectional. First, substance use may induce depressive symptoms [2]. Second, depressive symptoms may increase craving for a substance and use of the substance [34]. Therefore, a vicious cycle may lead to an association with more severe depression in dependent users than in non-dependent individuals.

The severity of depressive symptoms in the abstinent dependent ketamine users was higher than in the healthy controls and lower than in the current dependent ketamine users, which implied that depression did not remit even if the dependent ketamine users maintained abstinence. The gender difference in depression in the abstinent ketamine users with ketamine use disorder was significantly negative. A previous study showed that females are more likely to have depression and suffer from more severe depressive symptoms than males [35]. Our results further revealed that the difference in the severity of depression between genders in the abstinent ketamine users with ketamine use disorder was larger as compared with the healthy controls; this implied that female abstinent ketamine users may be associated with more severe depressive symptoms than male abstinent ketamine users. Meanwhile, the gender difference with regards to depression in the current ketamine users without ketamine use disorder was non-significant as compared with the gender difference in the healthy controls. This implied that the gender gap in regard to depression is more prominent in abstinent dependent ketamine users than in currently non-dependent ketamine users.

A previous review indicated that depression is associated with many substance-related symptoms and behaviors [36]. Furthermore, Deborah et al. conducted a study of patients with alcohol, cocaine and/or heroin dependence, and showed that depression can predict substance use and dependent relapse in those with sustained abstinence from drugs [37]. Therefore, depression may be an important issue for ketamine users, because depressive ketamine users may be more prone to continuing and re-starting substance use. Meanwhile, a study also revealed that some associations between depression and substance use behavior are gender-specific [36]. Our results further showed that the gender difference in depression varied by the status of ketamine use in ketamine users.

The QoL of the current ketamine users was lower than that of the healthy controls. This supported our hypothesis that current ketamine users have a lower QoL. Our results were in line with previous studies examining substances other than ketamine showing that current dependent users have a lower QoL than healthy controls [38,39]. The present study further showed that the non-dependent ketamine users also had a lower QoL. Furthermore, the dependent ketamine users had a lower QoL than the non-dependent ketamine users in the present study. Volk et al. demonstrated that dependent substance users have a lower QoL than non-dependent substance users [15]. Our results also indicated that patterns of current ketamine use may be associated with different levels of impairment of QoL.

The abstinent dependent ketamine users had a better QoL than the current dependent ketamine users, but a poorer QoL than the healthy controls; this may indicate that QoL may improve after they stopped ketamine use. Our results were also in line with a previous study that indicated an impaired QoL for abstainers in residential environments [40]. Many factors may be related to why the QoL of abstinent ketamine users is not as good as that of healthy controls [14]. Further study is warranted to explore how abstainers can be helped to return their QoL to a normal level. Females have a lower QoL throughout life than males in the general population [41,42,43]. Our results showed that the gender difference in QoL was reduced in the current ketamine users with and without ketamine use disorder. This indicated that the association between QoL and ketamine use is gender-specific.

The present study collected representative referents and conducted comprehensive control of confounding factors for comparison. Therefore, the results can provide clinicians with valuable information related to managing ketamine users. However, this study also had the following limitations. First, the WHOQOL-BREF is not a ketamine-specific instrument for the measurement of QoL; therefore, the QoL measures in the present study may not have been sensitive enough to reflect life quality in ketamine users. However, use of the WHOQOL-BREF to assess QoL has been validated for people in Taiwan [31,44]. Second, there may be other significant and unmeasured associations with QoL and depression in ketamine users. Further study is needed to explore other factors that may impact QoL and depression. Third, we cannot infer causality in observed associations with QoL and depression in view of the cross-sectional study design. Furthermore, longitudinal and interventional studies are warranted to explore the possible model for relationships between QoL and depression and ketamine use.

## 5. Conclusions

The guidelines for the management of substance use disorder with co-morbid depression consist of screening, regular assessment and interventions [45]. The current pharmacology suggestion for substance use disorder with co-morbid depression is antidepressants, especially for those with moderate and severe depressive symptoms. Furthermore, the SSRI remains the first choice for them [46]. In addition, decreasing substance use, maintaining abstinence and reducing psychosocial stress may be associated with improvements in the QoL for substance users [47,48,49]. In conclusion, patients with current ketamine use and those abstinent from ketamine use have more severe depressive symptoms and a reduced QoL across all domains. The poor QoL and greater severity of depression in patients with ketamine use highlight the need to address the affected domains of patients’ lives and their mental health during treatment in addition to focusing on their substance use. In order to support patients during their recovery process, clinicians need to be concerned with their QoL and be alert to depressive symptoms, and improvements in QoL and depression should be viewed as paramount in terms of achieving long-term clinical improvements and recovery. Furthermore, clinicians in this field need to address gender differences in QoL and depression when they evaluate and treat individuals with different status of ketamine use and provide an increased level of support for female ketamine users in order to achieve better improvement [50].

## Figures and Tables

**Table 1:** Severity of depression and quality of life (QoL) among healthy controls and subjects with differing ketamine use status.

Variables	Healthy Controls (*N* = 102)	Current Ketamine Users without Ketamine Use Disorder (*N* = 102)	Current Ketamine Users with Ketamine Use Disorder (*N* = 102)	Abstinent Ketamine Users with Ketamine Use Disorder in a Controlled Environment (*N* = 102)	*p*
	*N* (%) or Mean (SD)	*N* (%) or Mean (SD)	*N* (%) or Mean (SD)	*N* (%) or Mean (SD)	
Gender (male)	84 (82.35)	85 (83.33)	83 (81.37)	93 (91.18)	0.194
Age (years)	25.94 (5.30)	25.10 (5.29)	25.76 (5.97)	25.59 (6.79)	0.765
Education (years)	11.61 (2.03)	11.54 (2.82)	11.50(2.00)	11.06(2.02)	0.304
Severity of depression ^a^	13.49 (8.70)	17.21 (9.78)	25.47 (9.78)	18.83 (10.01)	<0.001
Physical domain of QoL ^b^	16.07 (1.74)	14.57 (1.87)	13.68 (1.65)	15.52 (2.25)	<0.001
Psychological domain of QoL ^b^	15.63 (2.37)	13.54 (1.68)	12.05 (1.40)	14.76 (2.45)	<0.001
Social domain of QoL ^b^	17.21 (2.21)	15.80 (2.32)	14.73 (2.28)	15.98 (2.66)	<0.001
Environmental domain of QoL ^b^	17.22 (1.54)	15.41 (2.22)	14.62 (1.76)	16.06 (2.34)	<0.001

^a^ as evaluated using the CES-D; ^b^ as evaluated using the WHO-QOL Brief.

**Table 2:** Gender differences in the severity of depression and quality of life (QoL) among healthy controls and ketamine-related groups.

	Depression		Physical Domain of QoL		Psychological Domain of QoL		Social Domain of QoL		Environmental Domain of QoL	
Variables	Mean (SD)	*p*	Mean (SD)	*p*	Mean (SD)	*p*	Mean (SD)	*p*	Mean (SD)	*p*
Healthy controls										
Female	21.72 (11.11)	<0.001	14.54 (1.61)	<0.001	13.15 (2.15)	<0.001	16.00 (2.09)	0.006	16.47 (1.29)	0.014
Male	11.70 (6.97)		16.39 (1.59)		16.17 (2.06)		17.46 (2.16)		17.38 (1.54)	
Current ketamine users without ketamine use disorder										
Female	17.71 (8.45)	0.368	14.96 (2.08)	0.345	13.33 (1.94)	0.699	15.94 (2.51)	0.812	16.47 (2.11)	0.019
Male	17.11 (10.06)		14.50 (1.83)		13.58 (1.64)		15.78 (2.29)		15.20 (2.19)	
Current ketamine users with ketamine use disorder										
Female	28.58 (10.87)	0.098	13.50 (1.67)	0.653	11.93 (1.66)	0.951	15.37 (2.09)	0.154	15.16 (1.41)	0.108
Male	24.76 (9.44)		13.72 (1.65)		12.07 (1.34)		14.58 (2.31)		14.50 (1.82)	
Abstinent ketamine users with ketamine use disorder										
Female	36.00 (7.91)	<0.001	13.62 (1.32)	0.004	12.74 (1.90)	0.009	14.67 (1.12)	0.027	14.62 (1.99)	0.031
Male	17.17 (8.55)		15.71 (2.24)		14.95 (2.41)		16.11 (2.74)		16.20 (2.33)	

**Table 3:** Associations of gender and status of ketamine use with the severity of depression and quality of life (QoL).

	Severity of Depression	Physical Domain of QoL	Psychological Domain of QoL	Social Domain of QoL	Environmental Domain of QoL
Variables	Coefficient	Coefficient	Coefficient	Coefficient	Coefficient
Gender ^a^	−7.15 ***	0.80 **	1.30 ***	0.38	0.01
Age (years)	0.07	−0.03	0.01	−0.01	−0.04
Education (years)	0.14	0.04	−0.01	−0.01	0.06
Status of ketamine use ^b^					
Abstinent ketamine users with ketamine use disorder in a controlled environment	6.08 ***	−0.60 *	−1.00 ***	−1.27 ***	−1.14 ***
Current ketamine users with ketamine use disorder	11.95 ***	−2.38 ***	−3.58 ***	−2.48 ***	−2.60 ***
Current ketamine users without ketamine use disorder	3.86 **	−1.52 ***	−2.11 ***	−1.42 ***	−1.84 ***

^a^ female as reference; ^b^ healthy controls as reference; * < 0.05; ** < 0.01; *** < 0.001.

**Table 4:** Interactions between gender and the status of ketamine use with the severity of depression and quality of life (QoL).

	Severity of Depression	Physical Domain of QoL	Psychological Domain of QoL	Social Domain of QoL	Environmental Domain of QoL
Variables	Coefficient	Coefficient	Coefficient	Coefficient	Coefficient
Gender ^a^	−10.00 ***	1.93 ***	3.01 ***	1.46 *	0.98 **
Age (years)	0.06	−0.03	0.003	0.01	−0.02
Education (years)	0.13	0.05	−0.002	−0.004	0.06
Status of ketamine use ^b^					
Abstinent ketamine users with ketamine use disorder in a controlled environment	14.27 ***	−0.72	−0.42	−1.36	−1.67 *
Current ketamine users with ketamine use disorder	6.98 *	−0.99	−1.22	−0.64	−1.25 **
Current ketamine users without ketamine use disorder	−3.74	0.44	0.19	−0.06	0.06
Interaction between gender and abstinent ketamine users with ketamine use disorder in a controlled environment	−8.82*	−0.08	−0.80	0.01	0.52
Interaction between gender and current ketamine users with ketamine use disorder	6.10	−1.70**	−2.87 ***	−2.25 **	−1.65 **
Interaction between gender and current ketamine users without ketamine use disorder	9.23 **	−2.40 ***	−2.77 ***	−1.62	−2.28 ***

^a^ female as reference; ^b^ healthy controls as reference; * < 0.05; ** < 0.01; *** < 0.001.

## Data Availability

The data will be available upon reasonable request to the corresponding authors.
